# Risk factors associated with cows’ lying time, stall and cows’ own cleanliness in smallholder dairy farms in Kenya

**DOI:** 10.14202/vetworld.2019.1085-1092

**Published:** 2019-07-21

**Authors:** E. K. Kathambi, J. A. VanLeeuwen, G. K. Gitau, C. Kamunde

**Affiliations:** 1 Department of Health Management, Atlantic Veterinary College, University of Prince Edward Island, Canada; 2 Department of Clinical Studies, Faculty of Veterinary Medicine, University of Nairobi, Kabete, Kenya

**Keywords:** dairy cows, Kenya, lying time

## Abstract

**Background and Aim:**

The welfare of animals kept in livestock production systems has raised concerns around the world. Adult dairy cattle require adequate rest and spend approximately 12 h/day lying down. This cross-sectional study aimed to determine the stall factors and management practices affecting cows’ lying time, stall cleanliness, and cows’ cleanliness (udder and upper leg), in smallholder dairy cows in Meru County of Kenya.

**Materials and Methods:**

A total of 106 milking cows from 73 farms were assessed for daily lying time and cleanliness. Data loggers were used to record the lying time of cows for 3 days. Stall, udder, and upper leg cleanliness were assessed using a 5-score system: 1 (very clean) to 5 (very dirty). Management information was acquired using a questionnaire that was administered face-to-face to the farmers in their native Kimeru language. Univariable and multivariable linear and logistic regression models were fit to determine factors associated with cows’ lying time and dichotomized stall and cows’ own cleanliness, respectively.

**Results:**

The mean daily lying time was 10.9±2.2 h, and the mean stall cleanliness score was 2.4±1.0. The mean average cleanliness scores of the udder and upper legs were 1.9±0.7 and 2.5±1.1, respectively. Overall, 35% of the stalls were categorized as dirty (>2.5), whereas 13% and 47% of the cows had udder and leg cleanliness scores >2.5, respectively. From the final multivariable models (p<0.05), daily lying time increased by 1.0 h for cows older than 5.25 years versus younger cows. Conversely, lying time decreased by 1.0 h with stall cleanliness scores >2.5 and by 1.6 h with poorly positioned neck rails. In an interaction term, addition of new bedding at least once a day without removing stall manure at least once a day decreased the daily lying time of the cows by 1.5 h, whereas failure to add new bedding at least once a day but removing stall manure at least once a day decreased the lying time of the cows by 1.2 h. Farm-level risk factors for stall dirtiness (>2.5) included delayed cleaning of the alley (odds ratio [OR]=6.6, p=0.032), lack of bedding (OR=4.9, p=0.008), and standing idle and/or backward in the stall (OR=10.5, p=0.002). Stalls categorized as dirty (OR=2.9, p=0.041) and lack of bedding (OR=2.7, p=0.065) were cow- and farm-level risk factors for dirtiness of the udder (>2.5), respectively, whereas the stall being dirty (OR=2.3, p=0.043) was the only risk factor (cow level) for dirtiness of the upper legs (>2.5).

**Conclusion:**

It was recommended that farmers should pay attention to the specific factors identified regarding the stall design (e.g., neck rail position) and bedding/manure management that impact the cleanliness of cows and their lying time.

## Introduction

The welfare of animals kept in livestock production systems has raised concerns around the world [1]. Some of the main cow welfare concerns include lying time, stall comfort and cleanliness, udder and leg cleanliness, mastitis, and lameness [2]. Adult dairy cattle require adequate rest and spend approximately 12 h/day lying down [3,4]. Video surveillance [5,6], motion sensors [7], and data loggers have been used to monitor cows’ behavior [8,9]. Poor comfort of dairy cows in industrialized countries leads to decreased lying time [10], reduced milk production [11], increased risk of lameness [12], and increased risk of mastitis [13].

Numerous cow- and stall-based factors affect cows’ lying time. Dairy cows’ lying times increase with an increase in parity [14,15] but decrease with increased milk production [8,16,17]. Cows spend more time lying down in stalls that have neck rails positioned higher above the floor surface [10,18], while more cows prefer stalls without a brisket board, especially if the stalls are short in length [19]. Cows prefer lying down on well-bedded surfaces [20,21] that are well maintained [22] and have dry bedding [10]. Conversely, the lying times of cows decrease as the quantity and quality (e.g., amount, depth, and dryness) of bedding decrease [23]. In addition, many of these cows- and stall-based factors also influence stalls’ and cows’ cleanliness [24,25]. Management practices such as frequency of manure removal have been shown to affect udder and leg cleanliness [26,27]. In Kenya, dairy cattle milk contributes about 70% of the total gross value of the livestock sector [28], with about 70% of the milk produced by smallholder dairy farmers (SDFs) [29]. However, little is known about the risk factors of comfort issues in dairy cows on SDFs in tropical countries such as Kenya [30,31].

The objective of this cross-sectional study was to assess the aspects of cows’ comfort and to determine stall design and management practices affecting lying time and stalls’ and cows’ cleanliness in smallholder dairy cows in Kenya.

## Materials and Methods

### Ethical approval

The study was approved by the Research Ethics Board and the Animal Care Committee of the University of Prince Edward Island, Canada, while the Naari Dairy Farmers Co-operativeSociety (NDFCS) in Kenya and Farmers Helping Farmers (FHF), a partnering non-governmental organization based in Canada endorsed the study.

### Informed Consent

The study was explained orally to all participants, and signatures for informed consent were obtained from all the participants in the study.

### Study design and sampling method

The study was carried out in the Naari region of Meru County in Kenya, where smallholder dairy farming is mainly practiced with zero-grazed and pasture-grazed farming systems. An initial simple random sample of 200 farms was computer generated from the list of 500 farmer members in the NDFCS. Of these 200 farms, 73 farms were selected to participate in this study, using the following inclusion criteria: (1) only farms with zero-grazing units; (2) up to four cows per farm; and (3) actively shipping milk to the NDFCS.

In each farm, an assessment of the stall design, stall cleanliness, and cow cleanliness was done on the first farm visit (see below). In addition, a questionnaire was administered face-to-face to acquire information on farm management practices. Data loggers (HOBO Pendant G Acceleration Data Logger [UA-004-64]; Onset Computer Corporation, MacArthur Blvd, Bourne, MA, USA) were attached to the left hind limb of each cow to determine their daily lying time. The specifications, calibration, and operation of the data loggers were done as per the manufacturer’s manual (HOBO Pendant G Acceleration Data Logger [UA-004-64] Manual). To ensure that the data loggers did not cause injury to the cows and were waterproof, they were wrapped in airtight disposable Ziploc^®^ bags. They were inserted into Velcro^®^ straps and attached on the inside of the left hind leg below the hock joint but above the fetlock joint, and 3 days after the first farm visit, the data loggers were removed, and the recorded data were transferred to a computer on the second farm visit.

### Data collection

Cow demographics, such as age and breed, were obtained in addition to daily milk yield per cow. The general health status and body condition score (1-5) [32] of the cows were established using physical examination, and lameness was assessed and categorized as absent, mild, or severe using a modified 5-point scoring system, collapsing scores 2 and 3 together, along with 4 and 5 [33].

For each stall, the length and width were measured and categorized as: (1) insufficient, (2) adequate, or (3) excessive, based on recommendations related to weight [34]. Availability and positioning of the neck rail and brisket board were assessed for height and distance from the rear curb [34] and categorized as: (1) present but not well-positioned, (2) present and well-positioned, or (3) not present in the stall. The lunge space and side leg space were assessed and categorized as: (1) insufficient space and/or railings present but in the wrong location; (2) appropriate space and railing positions; and (3) too much space and/or railings not present, based on recommendations [34].

Stall floor type was determined (dirt, wooden, or concrete), and floor flatness was assessed as flat (<5% if the floor uneven) or lumpy (≥5% if the floor uneven). The knee impact and knee wetness tests [35] were used to assess stall floors’ conditions. Availability of bedding on the lying surface, as well as the type of bedding used, such as sawdust, wood shavings, or crop waste, was determined (yes or no). The adequacy of the roof (yes or no) was determined based on a visual assessment that the roof was not allowing water to enter the stall. Adequate drainage of the stall (yes or no) was based on the gravitational flow of water along the alley floor. The condition of the alley was categorized based on the amount of manure at the time of assessment, where a clean alley had no manure, a fairly clean alley had small amounts of manure that could be easily avoided when walking in the alley, and a muddy alley had a large amount of manure that could not be avoided while walking. The stall, udder, and leg cleanliness scores were assessed using a 5-score system: 1 (very clean) to 5 (very dirty) [36]. For udder and leg cleanliness scores, an average between the left and right sides was recorded for the cows.

Data on farm-level parameters were acquired using a questionnaire that was administered to the farmers face-to-face by the investigator in the native language (Kimeru), which included: number of milking cows on the farm; frequency of hoof trimming; stall manure removal frequency; use of bedding on lying surfaces; frequency of adding new bedding in the stalls; and frequency of cleaning the alley. Abnormal lying and standing behaviors, such as perching, standing idle in the stall, standing backward in the stall, and lying in places other than the stall, were assessed while on the farm and/or reported by the farmers during the questionnaire interview.

### Data management and analysis

All data were entered, cleaned, and coded using Microsoft Excel^®^ 2013 (Microsoft, Sacramento, California, USA) and were analyzed using Stata 14.2^®^ (StataCorp, College Station, Texas, USA). Lying behavior data were analyzed in hours per cow per day. For continuous variables, means, medians, standard deviations (SDs), and ranges were used to describe the data. Proportions and their 95% confidence intervals were used to describe dichotomous variables such as lameness. Stall, udder, and leg cleanliness scores were: first described using means, SDs, and ranges; then dichotomized into binary outcomes (0=clean which included scores ≤2.5 and 1=dirty which included scores >2.5), and finally described using proportions and confidence intervals. Pearson’s correlation coefficient was used to assess any significant correlations (−0.35< r ≥0.35) between predictors to aid in model-building.

Univariable linear regression was used to determine unconditional associations of predictors with lying time at the cow level. Mixed multivariable linear regression models (p<0.05), with farm as a random effect to control for clustering of cows within farms, were also fit using the eligible predictors (p≤0.35), and confounders and two-way interacting variables were examined. Normality (Shapiro–Wilk test), homoscedasticity (Breusch–Pagan test), and linearity (scatter plots) were assessed for goodness-of-fit on the final multivariable linear regression models for lying time, whereas outlying and influential observations were assessed for the final model using standardized residuals, leverage, Cook’s distance, and delta-beta values.

For stalls’ and cows’ cleanliness, unconditional associations of predictors with these outcomes were initially assessed using univariable logistic regressions. Eligible factors (p≤0.35), confounders, and two-way interactions were fit into mixed multivariable logistic regression models (p<0.05). Goodness-of-fit tests (Hosmer–Lemeshow) were carried out for the multivariable logistic regression models. Outlying and influential observations were assessed for the final model using leverage and Cook’s distance values. The reliability of the model was assessed using the leave in and out protocol [37].

## Results

### Demographics of farms and cows

The 106 cows (1 to 4 cows per farm), on average (±SD), were 6±3 years old, weighed 363.5±55.4 kg, and had a body condition score of 2.4±0.4, with 94% of them categorized as predominantly exotic breeds and 6% as predominantly indigenous breeds. The mean daily milk yield per cow was 6.6±3.3 l, ranging from 1 to 21 l per cow per day. Three cows on three farms were currently being tethered outside instead of being kept in stalls at the time of the first visit, and therefore they were excluded from the remainder of the study. Of the 103 cows in stalls on 70 farms, 78% (80/103) had at least one of the abnormal behaviors: 33% of them (26/80) were standing idle and/or standing backward in the stall, whereas 67% (54/80) were lying down on other places (e.g., alley) and/or perching, in addition to either standing idle or standing backward in the stalls. No cows were categorized as lame in the study population.

### Stall descriptive and analytical statistics

For 13 cows, the farm did not have a complete stall; the partial stall was typically located in a corner of a pen, with a roof, a front wall, and one side wall to the stall. The average cleanliness score for the 90 complete stalls and 13 partial stalls was 2.4±0.9 and 2.8±1.2, respectively. Out of the 103 stalls, 36 (35%) were categorized as dirty (>2.5). The three stalls that had a well-positioned neck rail had a stall cleanliness score of 1 (very clean). The mean stall cleanliness score of stalls with a poorly positioned neck rail was 2.3, whereas the mean cleanliness score of stalls without a neck rail was 2.5.

Inadequate roofing and poor drainage were seen in 8% and 19% of the 103 stalls, respectively. A total of 79% of the 103 stalls had optimal length, whereas 26% had optimal width, based on the body weight of the cows. Neck rails, brisket boards, lunge space, and leg space were absent in 84%, 97%, 40%, and 26% of the 103 stalls, respectively. Of the 103 stalls, 90 (87%) had dirt floors, 62% (56/90) of which were categorized as lumpy. Thirteen stalls (12%) had concrete- or wooden-floored stalls, 38% (5/13) of which were considered lumpy. Sawdust or wood shavings were used as bedding in 33% (34/103) of the stalls, and crop waste was used in 39% (40/103) of the stalls, whereas 28% (29/103) of the stalls had no bedding. The knee impact and knee wetness tests failed in 13% and 11% of the 103 stalls, respectively. Out of 69 alleys assessed between the stalls and mangers (one missing data point), 39% (27/69) of them were classified as muddy and 40% (28/69) were fairly clean, with the remaining 21% being clean.

According to the farmers, stalls on 53% (37/69) of the farms had manure removed at least once a day, whereas alleys on 67% (46/69) of the farms were cleaned at least once a day. Nearly 72% (50/69) of the farms bedded stalls with sawdust, wood shavings, or crop waste and 56% (28/50) of these farms added new dry bedding to the stalls at least once a day. One farmhand could not answer these management questions, thus producing the missing data point.

Stall cleanliness had a strong correlation with wetness of the stall surface as determined using the knee test (r=0.8021). Univariable logistic regression analyses indicated that the following variables were associated with dichotomized stall dirtiness (p<0.35): availability of bedding, stall length, frequency of alley cleaning, abnormal cow behaviors, and presence and positioning of a neck rail.

A multivariable logistic regression model of stall dirtiness showed that bedding availability, frequency of cleaning the alley, and presence of abnormal resting behaviors in cows were statistically significantly (p<0.05) associated with dirty stalls in the final model. Failure to use any bedding on the lying surface increased the odds of stall dirtiness by 4.9 times (p=0.008). Delays to cleaning the alley (less than once a week) increased the odds of stall dirtiness by 6.6 times (p=0.032). Standing backward in the stall and idle standing and lying in places other than the stall increased the odds of stall dirtiness by 6.2 times (Table-1). In addition, stall length had a confounding effect on the association between the frequencies of alley cleaning and stall dirtiness. Thus, stall length was kept in the final model and was close to significant as well, with short stalls being protective against dirty stalls.

**Table 1:** Final multivariable logistic regression model of factors associated with dirty stalls used by 103 cows on 70 smallholder dairy farms in Kenya, 2017.

Factor	Categories	No. of cows	Odds ratio	95% CI	p-value
Bedding	Sawdust, wood shavings, or crop waste	74	Reference		
	None	29	4.97	(1.53, 16.15)	0.008
Frequency of alley cleaning	≥once/week	87	Reference		
	<once/week	16	6.63	(1.18, 37.35)	0.032
Abnormal behavior	None	23	Reference		0.007*
	Standing idle and/or standing backward	26	10.47	(2.31, 47.43)	0.002
	Standing idle, standing backward, lying on the alley, and/or perching	54	6.23	(1.63, 23.83)	0.008
Stall length	Optimal length	87	Reference		
	Too short	16	0.06	(0.01, 1.13)	0.060

* Overall p-value. CI=Confidence interval

### Cows’ lying time descriptive and analytical statistics

The median and mean (±SD) daily lying time of cows were 10.6 and 10.9±2.2 h, respectively, ranging between 2.9 and 19.0 h. Variables that had a significance level of p<0.35 in univariable linear regression analyses are shown in Table-2, with a comparison of lying times over the different levels of the predictor variables. Substantive correlations (−0.35<r>0.35) were stall cleanliness and knee wetness test of the lying surface (r=−0.802); adequate stall drainage and alley cleanliness (r=0.568); alley cleanliness and frequency of alley cleaning (r=0.496); and frequency of alley cleaning and frequency of stall manure removal (r=0.399).

**Table 2:** Descriptions and significance levels of differences in mean lying time in univariable analyses of lactating cows on smallholder farms in Kenya, 2017.

Factor	Categories	No. of cows	Lying time (hours)	

			Mean±SD	p-value
Cow-level variables (n=106 cows on 73 farms)				
Cows’ age (years)	≤5.25	53	10.46±2.06	0.024
	>5.25	53	11.42±2.24	
Body condition score	≤2.5	77	10.76±2.26	0.181
	>2.5	29	11.40±1.98
Stall-related variables (n=103 cows on 70 farms)				
Stall length	Optimal length	87	11.07±2.20	0.238
	Too short	16	10.36±1.97	
Neck rail positioning	Not available or well positioned	89	11.07±1.94	0.176
	Not well positioned	14	10.22±3.32	
Stall leg space	Available	76	11.18±2.21	0.077
	Not available	27	10.32±1.98	
Stall floor flatness	Flat	42	11.35±1.74	0.128
	Lumpy	59	10.69±2.40	
Stall drainage	Adequate	64	11.41±2.18	0.006
	Poor	39	10.20±1.97	
Stall wetness on knee test	Dry	42	11.47±2.18	0.048
	Wet	61	10.61±2.12	
Stall cleanliness	Clean (≤2.5)	67	11.35±2.17	0.011
	Dirty (>2.5)	36	10.22±2.01
Farm-level variables (n=103 cows on 70 farms)				
Abnormal behaviors	None	23	11.39±2.27	0.276
	Standing idle, standing backward, lying on the alley, and/or perching	80	10.83±2.14	
Alley cleanliness	Clean	65	11.38±2.06	0.009
	Muddy	38	10.23±2.19	
Frequency of manure removal	≥once a day	69	11.21±2.08	0.141
	<once a day	34	10.45±2.30	
Frequency of addition of new bedding	≥once a day	61	11.13±2.09	0.324
	<once a day	42	10.70±2.29	

SD=Standard deviation

**Figure-1:**
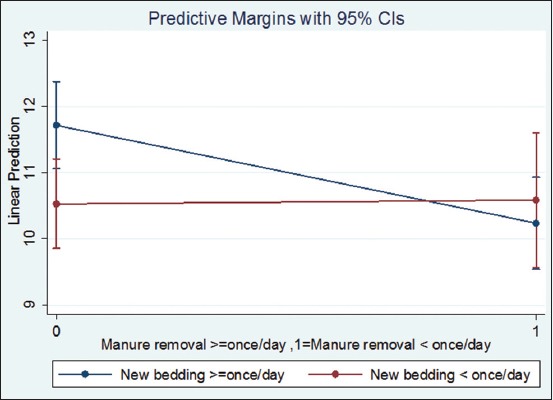
Interaction plot between frequency of stall manure removal and frequency of addition of new bedding on daily lying time of cows on 70 SDFs in Kenya.

The variability in the lying time outcome brought about by farm effects was negligible; hence, simple linear regression models with robust errors were preferred over mixed models. The final multivariable linear regression model of lying time indicated that age of the cow, neck rail positioning, stall cleanliness, frequency of manure removal, and frequency of adding new bedding were significantly associated with lying time. The lying time of cows older than 5.25 years was 1.0 h more than the lying time of cows younger than or equal to 5.25 years (Table-3). Stalls with poorly positioned neck rails resulted in cows lying down 1.6 h less compared with cows in stalls without a neck rail and those with a well-positioned neck rail. In stalls with cleanliness scores categorized as dirty (>2.5), cows spent 1.0 h less lying down per day, compared with cows in clean stalls. In a significant interaction variable, addition of new bedding at least once a day without removing stall manure at least once a day decreased the daily lying time of the cows by 1.5 h. Failure to add new bedding at least once a day but removing stall manure at least once a day decreased the lying time of the cows by 1.2 h. When both addition of new bedding and removal of stall manure were less than once a day, there was only a 1.1 h shorter lying time compared to when both addition of new bedding and removal of stall manure were at least once a day, but this category of the interaction was not statistically significant (Table-3 and Figure-1).

**Table 3:** Final multivariable linear regression model of factors associated with lying time of 103 cows from 70 smallholder farms in Kenya in 2017.

Factor	Coefficient	95% CI	p-value
Age (years)			
≤5.25	Reference		
>5.25	1.004	(0.318, 1.690)	0.005
Neck rail			
Not available or well positioned	Reference		
Not well positioned	−1.637	(−3.187, −0.087)	0.039
Stall cleanliness			
Clean	Reference		
Dirty	−0.969	(−1.676, −0.261)	0.008
Interaction variable for frequency of manure removal and frequency of addition of new bedding			
Manure removal and addition of new bedding≥once/day	Reference		0.040*
Manure removal≥once/day and addition of new bedding<once/day	−1.187	(−2.154, −0.221)	0.017
Manure removal<once/day and addition of new bedding≥once/day	−1.482	(−2.415, −0.550)	0.002
Manure removal and addition of new bedding<once/day	−1.134	(−4.498, 2.229)	>0.05

* Overall p-value. CI=Confidence interval

### Cows’ cleanliness descriptive and analytical statistics

The mean average udder and leg cleanliness scores for the 106 cows were 1.9±0.7 and 2.5±1.1, respectively, and ranged from 1 to 4 for the udder and 1 to 5 for the legs. A total of 13% and 47% of the cows had udders and legs categorized as dirty (>2.5), respectively.

The final multivariable logistic regression model for dirty udders showed that cows in stalls categorized as dirty (>2.5) had 2.9 times higher odds of having dirty udders (p=0.041) in comparison to cows in stalls categorized as clean (≤2.5), and failure to use any bedding on the lying surface increased the odds of udder dirtiness by 2.7 times, compared with using bedding (p=0.065). The final mixed logistic regression model of upper leg dirtiness found that cows in stalls categorized as dirty had higher odds (2.3 times) of having dirty upper legs (p=0.043) versus cows in stalls categorized as clean.

The final models all passed the respective goodness-of-fit tests for outlying and influential observations, residuals, normality, homoscedasticity, linearity, and the leave in and out protocol, where applicable. The final models for lying time, stall dirtiness, udder dirtiness, and leg dirtiness explained 21.1%, 21.6%, 9.7%, and 46% of the variation observed in stall dirtiness, lying time, udder dirtiness, and leg dirtiness, respectively.

## Discussion

Few studies on lying time, hygiene, and behavior of cows in smallholder dairy farms have been done in developing countries [30,31]. This is the first published article carried out on determining factors associated with lying time, stall cleanliness, and cow cleanliness on smallholder dairy farms in Kenya.

In this sample of smallholder dairy farms, the number of milking cows per farm was small (1.4 milking cows), which explains why the random farm effects anticipated while designing the study were essentially negligible. Daily milk yield of 6.6 kg/cow was lower than 9.3 kg/cow reported in the Mukurweini district of Kenya [30], which could be attributed to improved quality of feed, feeding practices, and other management practices on the farms in the Mukurweini district because there was a long-standing (over 10 years) cattle health management and development project in that area.

We found that shorter stalls had less likelihood of being dirty (Table-1) because the cows were likely to lie down in the alley rather than in the stall, leading to less contamination of the stall. In Norwegian farms [38], stalls that were too long allowed cows’ feces to fall inside the stall rather than the alley, increasing the likelihood of stalls being dirty.

Stalls without neck rails were more likely to be dirty in comparison to stalls with neck rails in our study (Table-1), and these findings are similar to those reported in Vancouver, British Columbia in 2005 [18]. Neck rails were absent in 84% of the 103 zero-grazing unit stalls; therefore, there is ample opportunity to improve stall cleanliness on smallholder dairy farms through proper neck rail installation.

Use of bedding materials, such as sawdust and wood shavings, improved stall cleanliness in the present study (Table-1) and also in Norwegian farms [39]. Bedding materials such as sawdust may have high moisture absorbency relative to dirt, wood, or concrete floors without bedding, thus improving stall cleanliness. In addition, good management practices, such as frequent removal of soiled bedding and addition of new dry bedding (at least daily), are important [26], and these were done in only 61% and 71% of the study farms, respectively.

Frequent cleaning of the alley was also associated with clean stalls in this study, and this finding is supported by similar findings that indicated improved stall cleanliness with cleaner alleys [27]. We observed that accumulated manure in the alley could be transferred to the stall by a cow’s feet during movement into the stall.

Abnormal cow behavior in stalls (e.g., perching and standing idle) is evidence of inappropriate stall design/management, and was found to be a significant variable in the final stall dirtiness model (Table-1). As anticipated, stall cleanliness scores were lower in farms that had cows exhibiting some of the abnormal behaviors such as standing backward in the stall and lying down in other places such as the alley.

The average cleanliness score of the upper legs was 2.5 using the 5-score system, whereas an average upper-leg cleanliness score of 2.9 was reported at a farm in Ontario, Canada, using the 4-score system [26]. The mean udder cleanliness score (1.9) in our study was also lower in comparison to findings from studies in Canada [26] and the Netherlands [13]. It is possible that cows in Canada and the Netherlands, with an average daily milk yield of 35.3 and 24.8 l, respectively, had larger udders that were more prone to getting soiled compared to the crossbred and indigenous cows in this study that had a daily milk yield of 6.6±3.3 l. The findings in our study that udders were cleaner than upper legs in the same cows were similar to those reported in cows in Ontario, Canada [40], likely due to the udders being cleaned prior to milking.

Udder dirtiness was associated with stall dirtiness and poor management practices, specifically failure to provide bedding in the stalls. Leg dirtiness was only associated with stall dirtiness and no other variables in the dataset. These findings are supported by results from various studies [26,27].

In the present study, cows spent an average of 10.9 h per cow per day lying down. This time was comparatively shorter than the 11.4 h per cow per day recorded in Ontario, Canada [26], and the 11.9 h per cow per day reported in Wisconsin, USA, in 2010 [6]. However, our daily lying time was much longer than the 9.0 h per cow per day reported in cows in Mukurweini, Kenya [30], which may reflect a group of farmers who were less informed on good cow comfort management. In our study, the shorter lying times compared with developed countries are likely due to suboptimal stall designs, availability of new bedding, and management practices, as found in our final multivariable model of lying time (Table-3). Addition of dry bedding on stalls without removing manure may negate the aim of keeping the stall dry, clean, and comfortable for the cows because moisture from the wet manure will seep easily to the new bedding, and this may explain the better lying time of cows receiving new bedding less than once a day but manure was removed at least once a day, when compared with cows on farms where new bedding was added at least once a day but manure was removed less than once a day (Table-3). The variation between cows in Canada and those in Kenya may be attributed to differing feeding intervals, housing systems, and management practices [41].

Lying time in our study increased with age, and this finding is consistent with findings from a study carried out on Holstein cows in Israel [42]. It is unclear why older cows might lie down more than younger cows. A higher prevalence of lameness and foot lesion reported in older cows has led to their increased lying time [43]. However, this association could not be assessed in our study due to the absence of observable clinical lameness.

We found that cows in stalls with poorly positioned neck rails spent less time lying down, in agreement with findings from a study carried out in French dairy farms [44]. However, these findings contrasted results from two studies that found no association between lying downtime and neck rail position [45,46]. A possible explanation for reduced lying time could be due to restricted movement during lying down and standing up by poorly positioned neck rails, which may make the cow prefer to stand rather than lie down, especially during the day time when the cows would be expected to feed at different times of the day.

With the high correlation between wetness and cleanliness of the stall (r=0.8021), we speculated that dirty stalls had a wet stall base and/or wet bedding, and therefore cows spent less time lying down in dirty stalls. A study carried out on dairy farms in Canada reported similar results and indicated that cows prefer dry stalls relative to wet stalls [23]. To ensure cleanliness of stalls, good stall management practices, including frequent manure removal and addition of new dry bedding, need to be carried out as shown in our final model.

One limitation of our study is the subjective nature of some assessments, such as udder and leg cleanliness scores and stall and alley conditions. Data collection was done by the principal investigator with the help of two veterinary students, and due to the impossibility of blinding the stall and cow cleanliness assessments, the subjectivity of some measures may have introduced some level of bias into those risk factor analyses. However, the lying time analyses would not be susceptible to lack of blinding bias because those data were objectively measured. To minimize this possible bias, during the training phase of the research project, the principal investigator and the two veterinary students all underwent the same training. Furthermore, consistency of assessments was examined and found to be good among the research team during the early part of the project, reducing the level of bias in the data due to subjectivity.

Another limitation of our study was that it was cross-sectional in nature, which prevents making conclusive causal statements regarding the observed associations due to the inherent lack of temporality between predictors and outcomes. Future research would benefit from a cohort study or randomized controlled trial to confirm the validity and importance of the observed factors associated with the various aspects of cow comfort involved in our study.

## Conclusions and Recommendations

One-third of the stalls assessed were categorized as dirty, over half of the cows had dirty hind legs, and the average lying time was suboptimal, demonstrating ample room for cow comfort improvement. From the final risk factor models, the key stall recommendations for farmers include ensuring properly installed neck rails; avoiding long stalls that allow manure deposits to fall into the stall; and addressing stall design features that lead to cows perching, standing idle or backward in the stall, or lying outside the stall (e.g., too wide or no lunge space). Key stall management recommendations include at least daily removal of manure from the stall; at least weekly removal of manure from the alley; and at least daily addition of new dry bedding to the stall. These recommendations should have a positive effect on the comfort and cleanliness of cows in smallholder farms in Kenya and other locations with similar circumstances, which lead to better performance and health of dairy cows.

## Authors’ Contributions

EKK, JAV, and GKG were involved in the study design, data collection, data analysis, and writing of the manuscript, while CK was involved in the study design, data analysis, and writing of the manuscript. All authors read and approved the final manuscript.

## References

[1] Rollin B.E (2004). Animal Agriculture and Emerging Social Ethics for Animals.

[2] Vasseur E, Gibbons J, Rushen J, Pellerin D, Pajor E, Lefebvre D, de Passillé A.M (2015). An assessment tool to help producers improve cow comfort on their farms. J. Dairy Sci.

[3] Jensen M.B, Munksgaard L, Pedersen L.J, Ladewig J, Matthews L (2004). Prior deprivation and reward duration affect the demand function for rest in dairy heifers. Appl. Anim. Behav. Sci.

[4] Jensen M.B, Pedersen L.J, Munksgaard L (2005). The effect of reward duration on demand functions for rest in dairy heifers and lying requirements as measured by demand functions. Appl. Anim. Behav. Sci.

[5] Cook N.B, Nordlund K.V, Oetzel G.R (2004). Environmental influences on claw horn lesions associated with laminitis and subacute ruminal acidosis in dairy cows. J. Dairy Sci.

[6] Gomez A, Cook N.B (2010a). Time budgets of lactating dairy cattle in commercial freestall herds. J. Dairy Sci.

[7] Lubaba C.H, Hidano A, Welburn S.C, Revie C.W, Eisler M.C (2015). Movement behavior of traditionally managed cattle in the eastern province of Zambia Captured using two-dimensional motion sensors. PLoS One.

[8] Bewley J.M, Boyce R.E, Hockin J, Munksgaard L, Eicher S.D, Einstein M.E, Schutz M.M (2010). Influence of milk yield, stage of lactation, and body condition on dairy cattle lying behavior measured using an automated activity monitoring sensor. J. Dairy Res.

[9] Ito K, Weary D.M, von Keyserlingk M.A.G (2009). Lying behavior:Assessing within and between-herd variation in free-stall-housed dairy cows. J. Dairy Sci.

[10] Fregonesi J.A, von Keyserlingk M.A.G, Weary D.M (2009). Cow preference and usage of free stalls compared with an open pack area. J. Dairy Sci.

[11] Uzal S, Ugurlu N (2010). The time budget of dairy cows as affected by season and housing system. J. Int. Environ. Appl. Sci.

[12] Dippel S, Dolezal M, Brenninkmeyer C, Brinkmann J, March S, Knierim U, Winckler C (2009). Risk factors for lameness in freestall-housed dairy cows across two breeds, farming systems, and countries. J. Dairy Sci.

[13] Dohmen W, Neijenhuis F, Hogeveen H (2010). Relationship between udder health and hygiene on farms with an automatic milking system. J. Dairy Sci.

[14] Sepélveda-Varas P, Weary D.M, von Keyserlingk M.A.G (2014). Lying behavior and postpartum health status in grazing dairy cows. J. Dairy Sci.

[15] Gomez A, Cook N.B (2010b). Time budgets of lactating dairy cattle in commercial freestall herds. J. Dairy Sci.

[16] Norring M, Valros A, Munksgaard L (2012). Milk yield affects time budget of dairy cows in tie-stalls. J. Dairy Sci.

[17] Miller-Cushon E, DeVries T.J (2017). Short communication:Associations between feed push-up frequency, feeding and lying behavior, and milk yield and composition of dairy cows. J. Dairy Sci.

[18] Tucker C.B, Weary D.M, Fraser D (2005). Influence of neck-rail placement on free-stall preference, use, and cleanliness. J. Dairy Sci.

[19] Tucker C.B, Zdanowicz G, Weary D.M (2006). Brisket boards reduce freestall use. J. Dairy Sci.

[20] Tucker C.B, Weary D.M (2004). Bedding on geotextile mattresses:How much is needed to improve cow comfort?J. Dairy Sci.

[21] Tucker C.B, Weary D.M, Fraser D (2003). Effects of three types of free-stall surfaces on preferences and stall usage by dairy cows. J. Dairy Sci.

[22] Drissler M, Gaworski M, Tucker C.B, Weary D.M (2005). Freestall maintenance:Effects on lying behavior of dairy cattle. J. Dairy Sci.

[23] Fregonesi J.A, Veira D.M, von Keyserlingk M.A.G, Weary D.M (2007). Effects of bedding quality on lying behavior of dairy cows. J. Dairy Sci.

[24] Fulwider W.K, Grandin T, Garrick D.J, Engle T.E, Lamm W.D, Dalsted N.L, Rollin B.E (2007). Influence of free-stall base on tarsal joint lesions and hygiene in dairy cows. J. Dairy Sci.

[25] Norring M, Manninen E, de Passillé A.M, Rushen J, Munksgaard L, Saloniemi H (2008). Effects of sand and straw bedding on the lying behavior, cleanliness, and hoof and hock injuries of dairy cows. J. Dairy Sci.

[26] DeVries T.J, Aarnoudse M.G, Barkema H.W, Leslie K.E, von Keyserlingk M.A.G (2012). Associations of dairy cow behavior, barn hygiene, cow hygiene, and risk of elevated somatic cell count. J. Dairy Sci.

[27] Magnusson M, Herlin A.H, Ventorp M (2008). Effect of alley floor cleanliness on free-stall and udder hygiene. J. Dairy Sci.

[28] Behnke R, Centre O, Wolford G, Muthami D (2013). A Living from Livestock IGAD Livestock Policy Initiative:The Contribution of Livestock to the Kenyan Economy. IGAD, Addis Ababa, Ethiopia.

[29] Rapsomanikis G, The Economic Lives of Smallholder Farmers (2015). An Analysis Based on Household Data from Nine Countries.

[30] Richards S.M (2017). Productivity and Welfare of Cows in Smallholder Dairy Farms in Kenya, Ph.D. Dissertation.

[31] Aleri J.W, Nguhiu-Mwangi J, Mogoa E.M (2011). Housing-design as a predisposing factor for injuries and poor welfare in cattle within smallholder units in Periurban areas of Nairobi, Kenya. Livest. Res. Rural. Dev.

[32] Wildman E.E, Jones G.M, Wagner P.E, Boman R.L, Troutt H.F, Lesch T.N (1982). A dairy cow body condition scoring system and its relationship to selected production characteristics. J. Dairy Sci.

[33] Sprecher D.J, Hostetler D.E, Kaneene J.B (1997). A lameness scoring system that uses posture and gait to predict dairy cattle reproductive performance. Theriogenology.

[34] Cook N.B (2009). Free-stall design for maximum cow comfort. WCDS. Adv. Dairy Technol.

[35] McFarland D.F (1991). Experiences with Free Stall Design in Pennsylvania. Paper American Society of Agricultural Engineers.

[36] Reneau J.K, Seykora A.J, Heins B.J, Endres M.I, Farnsworth R.J, Bey R.F (2005). Association between hygiene scores and somatic cell scores in dairy cattle. J. Am. Vet. Med. Assoc.

[37] Dohoo I.R, Martin S.W, Stryhn H (2012). Methods in Epidemiologic Research.

[38] Ruud L.E, Kielland C, Esterés O, Boe K.E (2011). Free-stall cleanliness is affected by stall design. Livest. Sci.

[39] Ruud L.E (2011). The Optimal Free Stall for Dairy Cows Effects of Free-stall Design on Cleanliness, Milk Yield, Health, and Behaviour.

[40] Zurbrigg K, Kelton D, Anderson N, Millman S (2005). Stall dimensions and the prevalence of lameness, injury, and cleanliness on 317 tie-stall dairy farms in Ontario. Can. Vet. J.

[41] Ito K, Chapinal N, Weary D.M, Von Keyserlingk M.A.G (2014). Associations between herd-level factors and lying behavior of freestall-housed dairy cows. J. Dairy Sci.

[42] Steensels M, Bahr C, Berckmans D, Halachmi I, Antler A, Maltz E (2012). Lying patterns of high producing healthy dairy cows after calving in commercial herds as affected by age, environmental conditions and production. Appl. Anim. Behav. Sci.

[43] Mason W (2017). Association between age and time from calving and reported lameness in a dairy herd in the Waikato region of New Zealand. N. Z. Vet. J.

[44] Veissier I, Capdeville J, Delval E (2004). Cubicle housing systems for cattle:Comfort of dairy cows depends on cubicle adjustment. J. Anim. Sci.

[45] Abade C.C, Fregonesi J.A, von Keyserlingk M.A.G, Weary D.M (2015). Dairy cow preference and usage of an alternative freestall design. J. Dairy Sci.

[46] Bernardi F, Fregonesi J.A, Winckler C, Veira D.M, von Keyserlingk M.A.G, Weary D.M (2009). The stall-design paradox:Neck rails increase lameness but improve udder and stall hygiene. J. Dairy Sci.

